# Effects of prophylactic administration of bacteriophages to immunosuppressed mice infected with *Staphylococcus aureus*

**DOI:** 10.1186/1471-2180-9-169

**Published:** 2009-08-17

**Authors:** Michał Zimecki, Jolanta Artym, Maja Kocięba, Beata Weber-Dąbrowska, Jan Borysowski, Andrzej Górski

**Affiliations:** 1 Institute of Immunology and Experimental Therapy, Polish Academy of Science, Wrocław, Poland; 2 Transplantation Institute, Medical University of Warsaw, Warsaw, Poland

## Abstract

**Background:**

Bacteriophages can be successfully applied to treat infections caused by antibiotic-resistant bacteria. Until now no attempts have been undertaken to treat infections in immunosuppressed patients with phages. In this work we investigated the prophylactic efficacy of specific bacteriophages in CBA mice treated with cyclophosphamide (CP) and infected with *Staphylococcus aureus*.

**Results:**

High numbers of bacterial colony-forming units in the organs as well as elevated tumor necrosis factor and interleukin-6 serum concentrations in CP-treated and *S. aureus*-infected mice were significantly lowered upon application of phages. The phages markedly increased the percentage of circulating neutrophils and immature cells from the myelocytic and lymphocytic lineages in CP-treated, *S. aureus*-infected mice as well as of myelocytes and immature neutrophils in the bone marrow. In addition, phages stimulated in such mice generation of specific agglutinins against *S. aureus*.

**Conclusion:**

Application of specific phages to immunosuppressed mice prior to infection with *S. aureus *proved very effective, suggesting a potential benefit of phage therapy in immunocompromised patients experiencing bacterial infections.

## Background

Phage therapy offers an excellent alternative to antibiotic therapy of bacterial infections (reviewed by [1]). Despite obvious efficacy in curing antibiotic-resistant infections it is still considered as "experimental" although it used to be a routine therapeutic approach to treat bacterial infections before introduction of antibiotics into therapy in the first half of the XX^th ^century. In contrast to antibiotics, which usually exhibit suppressive actions in relation to the immune response and deplete physiological intestinal microflora [2,3], the phage lytic action is highly selective. Moreover, phages demonstrate some bystander effects, beneficial to the function of the immune system such as: normalization of cytokine production by blood cells isolated from patients [4], acceleration of the neutrophil turnover [5], and inhibition of both bacteria- and LPS-induced respiratory burst by human blood phagocytes [6,7]. A discovery that phages may limit metastasis of B16 melanoma in mice [8] suggests a benefit of phage therapy in patients with malignant diseases.

Effectiveness of phage therapy may be, however, limited by several factors. Phage-resistant mutants has been observed in many phage-bacteria systems in Gram-positive and Gram-negative microorganisms [9]. Antibodies against bacteriophages may also appear during therapy [10,11]. Host specificity is another limitation. Majority of known bacteriophages are host-specific [12] and some are strain-specific [13]. Therapeutic phage preparations are mostly based on crude lyzates so they are not free from culture media ingredients and bacterial intracellular components including endotoxins. These agents are thought to be the reason of the adverse effects of phage therapy [14]. Lastly, a presence of lysogenic particles occurring in majority of bacterial population may also create a problem. In these cells bacteriophage genom is integrated within bacterial chromosome as prophage. Temperate bacteriophages may carry host genes to other bacterial cells and be responsible for horizontal gene transfer [15]. Nevertheless, despite all these limitations the phage therapy remains an alternative in antibiotic-resistant infections.

Although the majority of studies on phage therapy have been carried out on immunocompetent patients, there are also data indicating that phages could be effective and safe in immunocompromised individuals (for review see [16]). Of particular importance are the results achieved in immunocompromised cancer patients, which showed that phages could cure different kinds of bacterial infections without causing any serious side effects [17], as well as preliminary data obtained in a small group of renal transplant recipients (for references see [18]). Interestingly, phages may prolong mouse allograft survival, which constitutes an important argument for the safety of phage therapy in transplant recipients [19].

Although cyclosporine and steroids may not significantly impair function of cells responsible for innate immunity [20], some myeloablative agents like cyclophosphamide (CP) can transiently deplete the neutrophil pool [21] rendering a patient defenseless against infection. CP is widely used for treatment of autoimmune diseases [22-24] and leukemias [25]. The drug causes a profound, transient leukopenia [26], it also suppresses humoral [27] as well as cellular immune responses [28]. Although the neutropenia is transient and leads later to mobilization of myelopoiesis [29], the impairment of the specific humoral response, crucial for the development of adaptive immunity to pathogens, is long-lasting [27]. Therefore, the aim of this study was to evaluate effectiveness of prophylactic phage administration to CP-immunosuppressed mice on several parameters associated with innate and acquired immune response to *S. aureus *such as: number of bacteria in organs of infected mice, serum level of proinflammatory cytokines, blood and bone marrow cell profile and ability to generate specific antibody response to *S. aureus*.

In this work we convincingly demonstrate that administration of specific phages prior to infection can compensate the deficit of neutrophils in the clearance of *S. aureus *from the organs of CP-treated and infected mice. Moreover, the phages regulated the levels of proinflammatory cytokines and elicited mobilization of cells from both myelocytic and lymphocytic lineages. Lastly, the application of phages stimulated generation of specific antibodies to *S. aureus *and to an unrelated antigen sheep red blood cells.

## Methods

### Mice, strains and reagents

CBA male mice, 10–12 weeks-old, were purchased from Ilkowice/Kraków, Poland. The mice had free access to water and standard rodent laboratory chow. All protocols were approved by the local ethics committee.

*Staphylococcus aureus *L strain was isolated from a 26-year old patient A.L., suffering from pharyngitis. *In vitro *analysis (antibiogram) revealed its sensitivity to the following antibiotics: amikacin, ciprofloxacin, doxycycline, erythromycin, gentamycin, clindamycin and methicillin. Unfortunately, the antibiotic treatment was not effective so the patient was subsequently subjected to successful phage therapy. This is a typical *S. aureus *strain producing beta-hemolysin. The lethal dose of this strain for CBA mice pretreated with 350 mg/kg b.w. of CP was 4 × 10^8 ^(LD100). Both *S*. *aureus *strain and *S. aureus *A5/L bacteriophages are deposited in the Bacteriophage Laboratory of the Institute of Immunology and Experimental Therapy, Wrocław. The preparation and purification of specific bacteriophages were described by us elsewhere [30]. LPS contamination of the phage preparation was negligible as determined by Limulus amebocyte lysate (LAL) (1.8 E.U. per 10^6 ^phages). Cyclophosphamide (CP) was from ASTA Medica, Frankfurt, Germany.

### Treatment of mice with cyclophosphamide, *S. aureus *and bacteriophages

Mice were injected with CP (200 or 350 mg/kg b.w.) intraperitoneally (i.p.) as indicated in the figure legends. Bacteria were administered intravenously (i.v.), into lateral tail vein, four days after CP, at a dose of 5 × 10^6^/mouse. Bacterial cell numbers were determined colorimetrically at a wavelength of 600 nm according to previously prepared standards. Virulent *S. aureus *A5/L bacteriophages were administered i.p. 30 minutes before infection, at a dose of 1 × 10^6^/mouse. Control mice received 0.2 ml of 0.9% NaCl instead of bacteria and phages. In some experimental protocols control mice were given phages or bacteria only.

### Determination of *S. aureus *in the organs

Twenty four hours after infection, the mice were sacrificed, the organs (spleens, livers and kidneys) were isolated and homogenized using a plastic syringe piston and a plastic screen, in sterile PBS (1 g of wet tissue per 25 ml of PBS). Five- and fifty-fold dilutions of cell suspension were applied onto Chapmann agar plates and incubated overnight and the colony-forming units (CFU) were enumerated. The number of colonies was expressed as the number of CFU per milligram of the organ.

### Analysis of cell types in the circulating blood and bone marrow

Samples of blood were taken on day 0, just before administration of CP, 4 days after administration of CP, just before administration of phages and bacteria (day 4) and at 24 h following infection (day 5). The bone marrow was isolated on days 0 and 5. Blood and bone marrow smears were prepared and stained with May-Grünwald and Giemsa reagents. The preparations were reviewed microscopically by a histologist at 1000× magnification.

### Determination of serum TNF-α and IL-6 levels

The activities of TNF-α and IL-6 in sera were determined by bioassays using WEHI 164.13 and 7TD1 cell lines, respectively [31,32].

### Determination of serum antibody titer to *S. aureus *and sheep red blood cells (SRBC)

Mice were given CP (200 mg/kg b.w.). After four days the mice were infected i.v. with *S. aureus *at a dose of 5 × 10^6^/mouse and administered i.p. A5/L phage (1 × 10^6^/mouse). 21 days later the mice were bled and the sera isolated. Using a similar protocol mice were immunized with 0.2 ml of 10% SRBC i.p., one hour after infection with *S. aureus *and the mice were bled on day 10 after immunization. The agglutination test for measuring the titer of anti-*S. aureus *antibodies was performed as follows: 50 μl of two-fold sera dilutions were distributed in 96-well microtiter plates and 25 μl of 1% thermally-inactivated *S. aureus *suspension was added. After 1 h incubation at room temperature the agglutination was determined in a microscope. The hemagglutination test was performed analogously using 1% SRBC suspension as antigen.

### Statistical analysis

The results of one representative experiment, out of three performed, were shown. For statistical evaluation of the data, analysis of variance (ANOVA) or ANOVA of Kruskal-Wallis as well as *post hoc *tests were applied. The Brown-Forsyth's test was used to determine the homogeneity of variance. Depending on type of experiment groups consisted of 5–15 mice. The results are presented as mean or median values and were regarded to be significant when P < 0.05. Only significant and relevant comparisons described in the Results section were shown. The name of groups in the text and figure legends are designated as follows: CP^+^P^+^B^+ ^(mice treated with: cyclophosphamide, phages, and bacteria, respectively), CP^+^P^-^B^+ ^(mice represent a group of animals pretreated with CP, infected with bacteria but not given phages).

## Results

### Effect of bacteriophages on the clearance of *S. aureus *in organs of infected mice, serum IL-6 and TNF-α levels and titer of anti-*S. aureus *agglutinis

**Figure 1:**
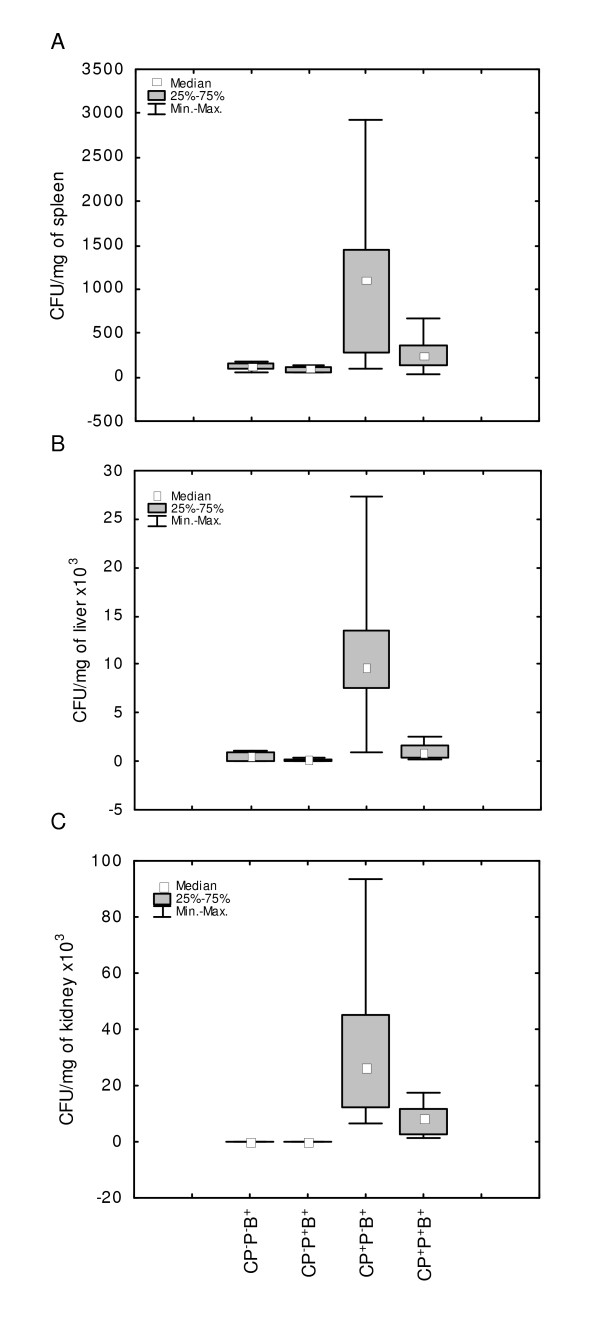
**Protective effect of A5/L phages on *S. aureus *infected mice pretreated with cyclophosphamide**. **A**: spleen, **B**: liver, **C**: kidney. Mice were given CP (350 mg/kg b.w.). After four days A5/L phages (10^6^) were administered 30 minutes before infection of mice with 5 × 10^6 ^of *S. aureus*. 24 h later the CFU were enumerated in the organs. The number of mice per group: n = 20. Statistics: **A: **CP^-^P^-^B^+ ^*vs *CP^+^P^-^B^+ ^*P *= 0.0004; CP^+^P^-^B^+ ^*vs *CP^+^P^+^B^+ ^*P *= 0.0169 (ANOVA of Kruskal-Wallis; *P *= 0.0000); **B: **CP^-^P^-^B^+ ^*vs *CP^+^P^-^B^+ ^*P *= 0.0004; CP^+^P^-^B^+ ^*vs *CP^+^P^+^B^+ ^*P *= 0.0009 (ANOVA of Kruskal-Wallis; *P *= 0.0000); **C: **CP^-^P^-^B^+ ^*vs *CP^+^P^-^B^+ ^*P *= 0.0001; CP^+^P^-^B^+ ^*vs *CP^+^P^+^B^+ ^*P *= 0.0370 (ANOVA of Kruskal-Wallis; *P *= 0.0000).

Mice were treated with CP, bacteriophages and infected with bacteria as described in the Materials and Methods. Control mice received no phages. 24 h after the infection the bacteria numbers were enumerated in spleens, livers and kidneys. Mice not treated with CP served as additional controls. The results shown in Figure 1 indicate that highly elevated CFU numbers in CP-treated mice (CP^+^P^-^B^+^) were lowered by the application of phages (CP^+^P^+^B^+ ^mice) to the values observed in mice not subjected to CP treatment (CP^-^P^-^B^+ ^group).

**Figure 2:**
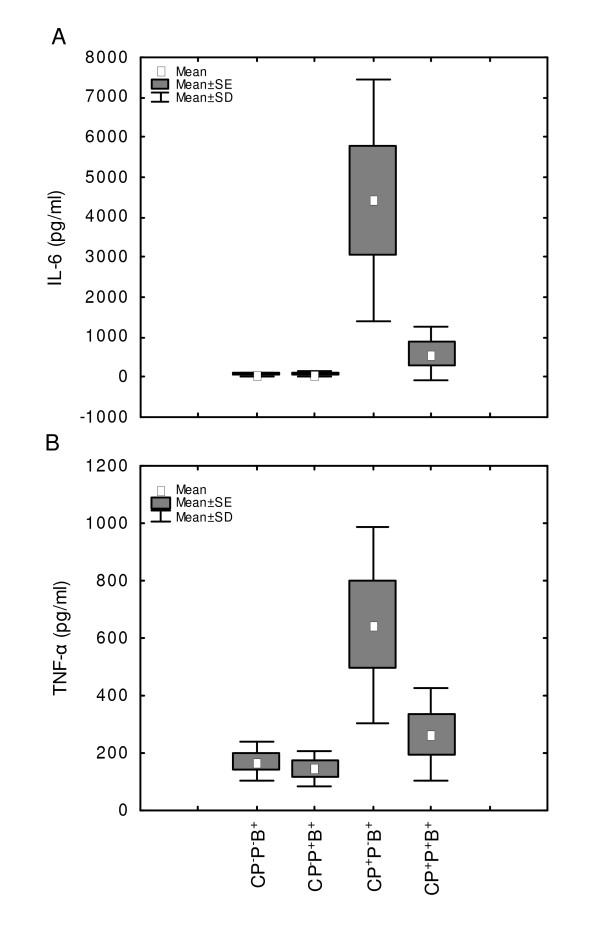
**Effects of A5/L phages on IL-6 and TNF-α serum levels in cyclophosphamide-treated and *S. aureus*-infected mice**. **A**: IL-6, **B**: TNF-α. Some mice from the experiment described in Figure 1 were bled for cytokine determination at 24 h following infection. The number of mice per group: n = 5. Statistics: **A: **CP^-^P^-^B^+ ^*vs *CP^+^P^-^B^+ ^*P *= 0.0023; CP^+^P^-^B^+ ^*vs *CP^+^P^+^B^+ ^*P *= 0.0064 (ANOVA; *P *= 0.0009); **B: **CP^-^P^-^B^+ ^*vs *CP^+^P^-^B^+ ^*P *= 0.0065; CP^+^P^-^B^+ ^*vs *CP^+^P^+^B^+ ^*P *= 0.0301 (ANOVA; *P *= 0.0028).

The elevated IL-6 concentrations at 24 h after infection in CP-treated (CP^+^P^-^B^+^) mice (4427 pg/ml *versus *80 pg/ml in control CP^-^P^-^B^+ ^mice) were significantly lowered (*P *= 0.0064) upon phage application (593 pg/ml) (Figure 2A). Similarly, elevated TNF-α concentrations in CP-treated mice (647 *versus *170 pg/ml in control CP^-^P^-^B^+ ^mice) were significantly (*P *= 0.0301) decreased by the administration of phages (264 pg/ml) (Figure 2B).

### Effects of bacteriophages on cell composition in circulating blood and bone marrow

**Figure 3:**
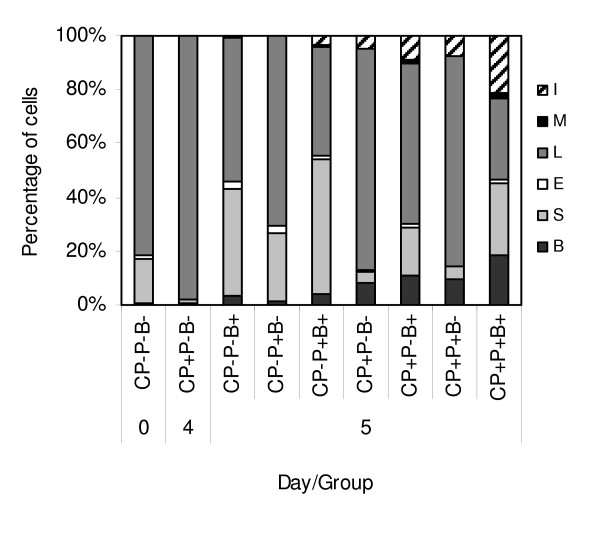
**Effects of A5/L phages on the circulating blood cell composition in cyclophosphamide-treated and *S. aureus*-infected mice**. B – bands, S – segments, E – eosinophils, L – lymphocytes, M – monocytes; I – immature forms. Mice were given CP (350 mg/kg b.w.). After four days 1 × 10^6 ^A5/L phages and 5 × 10^6 ^*S. aureus *were administered. Samples of blood were taken on day 0, just before administration of CP (Control), 4 days after administration of CP, just before administration of phages and bacteria (day 4) and at 24 h following infection (day 5). The results are presented as the mean value of 5 mice per group. Statistics (day 5): **Bands: **CP^+^P^-^B^+ ^*vs *CP^+^P^+^B^+ ^*P *= 0.0261 (ANOVA; *P *= 0.0000); **Segments**: CP^-^P^-^B^+ ^*vs *CP^+^P^-^B^+ ^*P *= 0.0003; CP^+^P^-^B^- ^*vs *CP^+^P^-^B^+ ^*P *= 0.0489 (ANOVA; *P *= 0.0000); **Eosinophils: **all crucial comparisons NS (ANOVA); **Lymphocytes: **CP^+^P^-^B^+ ^*vs *CP^+^P^+^B^+ ^*P *= 0.0003; CP^+^P^-^B^- ^*vs *CP^+^P^-^B^+ ^*P *= 0.0042 (ANOVA; *P *= 0.0000); **Monocytes: **all crucial comparisons NS (ANOVA); **Immature forms: **CP^-^P^-^B^+ ^*vs *CP^+^P^-^B^+ ^*P *= 0.0498 (ANOVA; *P *= 0.0000).

In order to evaluate effects of phage application on contribution of cells involved in non specific antimicrobial defense of CP-immunocompromised and *S. aureus*-infected mice, we determined alterations in the cell composition of the circulating blood and bone marrow. Alterations in the cell composition of the circulating blood on day 5 in relation to CP treatment, 24 h following infection and administration of phages, are presented in Figure 3. Although in infected, not CP-treated mice, the changes in the blood cell composition induced by phages were not significant, we found them more profound in CP-treated mice. First, in CP^+^P^-^B^+ ^mice, apart from mature neutrophils, a fraction of immature neutrophils (bands) and more immature cells (undifferentiated cells, myelocytes, metamyelocytes and lymphoblasts) appeared, although on day 4 following CP administration, just before infection, such cells were virtually not existing in the circulation. Secondly, the administration of phages (CP^+^P^-^B^+ ^*versus *CP^+^P^+^B^+ ^group) significantly enlarged the content of band forms (*P *= 0.0261).

**Figure 4:**
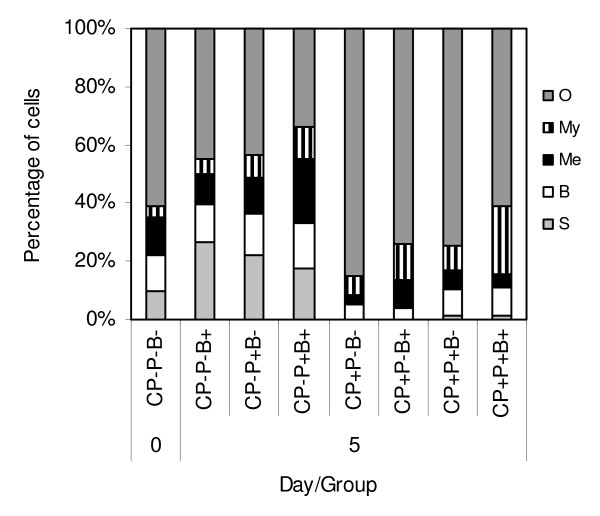
**Effects of A5/L phages on the bone marrow cell composition in cyclophosphamide-treated and *S. aureus*-infected mice**. S – segments, B – bands, Me – metamyelocytes, My – myelocytes, O – other. Mice were given CP (350 mg/kg b.w.). After four days 1 × 10^6 ^A5/L phages and 5 × 10^6 ^*S. aureus *were administered. The bone marrow was isolated on day 0, just before administration of CP (Control) and at 24 h following infection (day 5). The results are presented as the mean value of 5 mice per group. Statistics (day 5): **Segments: **CP^-^P^-^B^+ ^*vs *CP^+^P^-^B^+ ^*P *= 0.0001 (ANOVA; *P *= 0.0000); **Bands: **CP^-^P^-^B^+ ^*vs *CP^+^P^-^B^+ ^*P *= 0.0009; CP^+^P^-^B^+ ^*vs *CP^+^P^+^B^+ ^*P *= 0.0495 (ANOVA; *P *= 0.0000); **Metamyelocytes: **CP^-^P^-^B^+ ^*vs *CP^-^P^+^B^+ ^*P *= 0.0003 (ANOVA; *P *= 0.0000); **Myelocytes: **CP^+^P^-^B^+ ^*vs *CP^+^P^+^B^+ ^*P *= 0.0062 (ANOVA; *P *= 0.0000); **Other: **CP^-^P^-^B^+ ^*vs *CP^+^P^-^B^+ ^*P *= 0.0003 (ANOVA;*P *= 0.0000). Statistics (day 0 *vs *day 5): **Segments: **CP^-^P^-^B^- ^*vs *CP^-^P^-^B^+ ^*P *= 0.0001; CP^-^P^-^B^- ^*vs *CP^-^P^+^B^- ^*P *= 0.0000 (ANOVA); Metamyelocytes: CP^-^P^-^B^- ^*vs *CP^-^P^+^B^+ ^*P *= 0.0000 (ANOVA); **Myelocytes: **CP^-^P^-^B^- ^*vs *CP^-^P^+^B^+ ^*P *= 0.0016 (ANOVA).

The alterations in the bone marrow cell type composition of mice from the same experiment are presented in Figure 4. The infection of control mice (CP^-^P^-^B^+ ^*versus *CP^-^P^-^B^-^) led to an increase of the segments content (*P *= 0.0001) and co-administration of phages (CP^-^P^+^B^+ ^group) markedly increased the percentage of myelocytes (*P *= 0.0016) and metamyelocytes (*P *= 0.0000). In CP-treated and infected mice (CP^+^P^-^B^+^) there was a deficit of bands and no segments were present, however application of phages in these mice (CP^+^P^+^B^+ ^group) led to a significant (a two-fold) mobilization of myelocytes (*P *= 0.0068) and bands (*P *= 0.0495). Interestingly, the phages alone (CP^-^P^+^B^-^) increased (*P *= 0.0000) the content of segments in control, not infected mice (CP^-^P^-^B^-^). Other changes following phage administration were not significant.

### Effects of the phages on generation of the humoral response to *S. aureus *and to sheep erythrocytes

**Figure 5:**
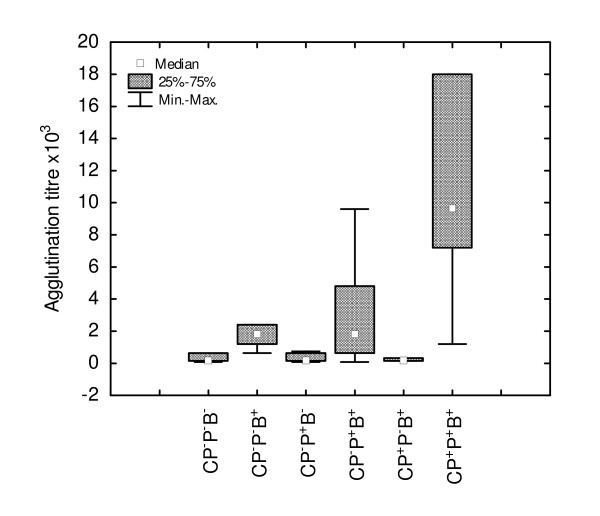
**Enhancing effect of A5/L phages on *S. aureus*-specific antibodies in cyclophosphamide-treated and infected mice**. Mice were given CP (200 mg/kg b.w.). After four days the phages 1 × 10^6 ^and bacteria (5 × 10^6^) were administered. The mice were bled for the measurement of antibody titer 21 days later. The number of mice was 10 per group. Statistics: CP^-^P^-^B^- ^*vs *CP^-^P^-^B^+ ^*P *= 0.0495; CP^-^P^-^B^+ ^*vs *CP^+^P^-^B^+ ^*P *= 0.0369; CP^+^P^-^B^+ ^*vs *CP^+^P^+^B^+ ^*P *= 0.0001 (ANOVA of Kruskal-Wallis; *P *= 0.0000).

A possibility existed that phages, beside their direct, protective role during infection, may stimulate generation of specific immune response against bacteria. Figure 5 shows the effects of phage administration on the agglutinin level in mouse sera taken 21 days following intraperitoneal immunization of mice with 5 × 10^6 ^of *S*. *aureus *(for details see Materials and Methods). The results revealed a strong up-regulation (*P *= 0.0001) of anti-*S*. *aureus *agglutinin titer in CP and phage-treated mice (CP^+^P^+^B^+^) in comparison with a respective control (CP-treated mice) (CP^+^P^-^B^+ ^group). The analogous effect of phages in mice not treated with CP was minor (CP^-^P^+^B^+ ^*versus *CP^-^P^-^B^+ ^group). The phages also enhanced (not significantly), the titer of hemagglutinins to SRBC in CP-treated and infected mice (data not shown).

## Discussion

The results presented in this report demonstrated not only efficient removal of the bacterial load in infected mice virtually devoid of major functional phagocytes, by prophylactic administration of specific phages, but also revealed accompanying, beneficial effects on the immune system, mediated by *S. aureus *phage preparation in the described model. It appeared that application of phages in infected mice may accelerate renewal of cells depleted by CP treatment, both of the myelocytic and lymphocytic lineages. The first type of cells has significance in the first-line defense against bacteria as phagocytes and the latter differentiate to mature, immunocompetent cells, giving rise to adaptive, antigen-specific immune response.

It is conceivable that the stimulation of hematopoiesis by phages is initiated by destruction of bacterial walls and release of bacterial antigens acting as adjuvants for the immune system. The stimulatory effects of different bacterial antigens on hematopoiesis in CP-immunosuppressed mice were reported by others [33,34]. The increased stimulation of hematopoiesis in infected, phage-treated (CP^+^P^+^B^+^) mice *versus *infected (CP^+^P^-^B^+^) mice or mice treated only with phages (CP^+^P^+^B^-^), found in this study, supports such a notion. In infected mice not treated with CP, the phages elevated the percentage of mature neutrophils (segments) (CP^-^P^+^B^+ ^*versus *CP^-^P^-^B^+ ^mice), although not significantly. That phenomenon could represent an additional output of mature neutrophils from the bone marrow reservoir which is particularly large in rodents [35]. The infection of CP-treated mice (CP^+^P^-^B^+ ^group) resulted in a characteristic change in the blood picture with appearance of immature and mature neutrophils as well as more immature cells from myelo- and lymphocytic lineages. The proportion of these cell types, including a small contribution of eosinophils and monocytes, significantly increased in mice treated additionally with phages (from 40.4 to 70.2%) (Figure 3). The effective killing of bacteria in the investigated organs, particularly in the liver, where most of the killing takes place [36], probably resulted from the increased number of phagocytes, as shown in this work, although we assume that a contribution of phages in that process is the major one.

The bone marrow picture in normal mice showed a significant increase of the mature neutrophils content after infection (CP^-^P^-^B^+ ^group), with a reduction in these cells upon phage application that suggests an accelerated export of neutrophils into periphery. The loss of neutrophils in the bone marrow was, in this case, compensated by an enlargement of the metamyelocyte pool (Figure 4). Interestingly, the application of phages alone (CP^-^P^+^B^- ^mice) led also to some increase of the neutrophil cell content. However, it cannot be excluded that even well-purified phage preparations used in our experiments still contain some components of bacterial cells, which could contribute to the induction of myelopoiesis. Although the administration of CP (CP^+^P^-^B^- ^mice) caused an anticipated profound loss of the neutrophil cell lineage, the infection (CP^+^P^-^B^+ ^mice) enlarged the fractions of myelocytes and metamyelocytes. The administration of phages (CP^+^P^+^B^+ ^mice), however, doubled the proportion of myelocytes (from 12.4 to 23.4%) and bands (from 4.0 to 9.8%). The significant increase of the myelocyte pool in the bone marrow suggests that phages recruit this cell type from more immature precursors. In addition, phage preparations apparently support the transition of metamyelocytes to band forms (Figure 4). Taking these observations together, we may conclude that phages in infected, CP-immunosuppressed mice act at various stages of the myeloid cells differentiation, promoting both the recruitment of the immature neutrophil cell types from their precursors in the bone marrow and triggering more rapid output of mature functional neutrophils into periphery.

We can not exclude involvement of other cells capable of removing bacteria from the circulation, which could be spared following CP administration such as monocytes and macrophages residing in the peritoneal cavity and organs of the reticuloendothelial system, in particular Kupffer cells [36,37]. Nevertheless, the role of Kupffer cells in the process of bacteria clearance seems to be auxiliary for neutrophils [37] which are regarded as the major phagocyte cell type.

Although we have collected, in the past, observations regarding acquisition of specific immunity by patients following successful phage therapy, no scientific documentation exists to support such findings. In this study we showed that administration of specific phages during experimental infection, in particular in CP-treated mice, led to a higher titer of *S. aureus *serum agglutinins in comparison with respective controls (Figure 5). That phenomenon was accompanied by the appearance of lymphoblasts in circulation indicating that the phages may elicit lymphopoiesis in the bone marrow. Although CP is cytotoxic, particularly for B cells [38], it spares stem cells [39] which may serve as a source of a new generation of immunocompetent T and B cells. Because of high toxicity of CP in relation to B cells we applied in this experiment a somewhat lower (200 mg/kg b.w.) dose of the drug still, however, able to significantly suppress the humoral immune response [40]. The CP-treated mice were also able to mount an increased, specific immune response to an unrelated antigen SRBC. That phenomenon supported our assumption that the increase of immune system potential was a consequence of a bystander (adjuvant) effect of bacterial destruction by phages.

Neutropenic mice display elevated cytokine levels after infection [41] that was also confirmed in this study. The inhibitory effects of phages on bacterial CFU numbers in CP-treated and infected mice (CP^+^P^+^B^+ ^group) were associated with diminished serum levels of pro-inflammatory cytokines. This phenomenon could be interpreted as a profoundly decreased necessity to ingest bacteria by phagocytes due to removal (lysis) of bacteria by phages. In such a case release of proinflammatory cytokines which occurs upon phagocytosis [42] would be diminished. The down-regulatory effects of phages on the levels of pro-inflammatory cytokines (particularly TNF-α) during bacterial infection (Figure 2), are in contrast to apparently harmful, increased production of TNF-α during infection induced by antibiotics [43-45]. Anti-TNF-α antibody can reduce mortality of mice during antibiotic-induced TNF-α release during infection [45], providing a proof for the lethal effects of TNF-α. In the case of *S. aureus*, beta-lactam antibiotics increased release of TNF-α in culture of mouse peritoneal macrophages and the inducing factor was identified as protein A [44]. It is, therefore, likely that the lytic action of A5/L bacteriophages leads to a much lesser exposure of bacterial cell components to cells of the immune system.

Administration of phages shortly before infection is a limitation of this model since it does not reflect a therapeutical approach. We intend to extend the studies on immunocompromised mice using a delayed phage application.

## Conclusion

In summary, this is to our knowledge the first study in a mouse experimental model showing that prophylactic phage administration proved both safe to the immunosuppressed mice and seemed to serve as immune-function replacement role. The mobilization of myelopoiesis and stimulation of the specific, protective antibody response was a basis for the successful application of phages in these mice. These results suggest not only safety but also beneficial effects of phage therapy on the immune status of immunosuppressed patients.

## Competing interests

The authors declare no conflict of interest except of AG and BWD who have pending patent application for preparation of *S. aureus *phages.

## Authors' contributions

ZM designed the experiments and prepared the manuscript. AJ participated in performing the experiments and was responsible for preparing figures and statistical evaluation. KM participated in performing experiments and preparation of data. W-DB was responsible for supplying bacteria and bacteriophage preparations, BJ and GA participated in preparation of the manuscript and served as consultants. All authors read and approved the final manuscript.
